# Evaluation of the diagnostic ability of laminin gene family for pancreatic ductal adenocarcinoma

**DOI:** 10.18632/aging.102007

**Published:** 2019-06-10

**Authors:** Chengkun Yang, Zhengqian Liu, Xianmin Zeng, Qiongyuan Wu, Xiwen Liao, Xiangkun Wang, Chuangye Han, Tingdong Yu, Guangzhi Zhu, Wei Qin, Tao Peng

**Affiliations:** 1 Department of Hepatobiliary Surgery, The first Affiliated Hospital of Guangxi Medical University, Nanning, Guangxi Province, China; 2 Department of Tuina, The First Affiliated Hospital of Guangxi University of Chinese Medicine, Nanning, Guangxi Province, China

**Keywords:** PDAC, laminin, CA19-9, prognosis, diagnosis

## Abstract

A poor outcome for pancreatic ductal adenocarcinoma (PDAC) patients is still a challenge worldwide. The aim of our study is to investigate the potential of key laminin subunits for being used both as a diagnostic and prognostic biomarker for PDAC patients. We evaluated the mRNA expression and prognostic value of laminin gene family in PDAC tissues using online public databases. Moreover, the relationship between key laminin subunits in PDAC blood cells and circulating tumor cells (CTCs) and the distinguishing ability of joint serum levels with carbohydrate antigen 19-9 (CA19-9) was analyzed. Two key differentially expressed subunits (LAMA3 and LAMC2) that are associated with prognosis of PDAC patients were found to show a potential for distinguishing between PDAC and non-tumor tissues. LAMA3 and LAMC2 expression were found to be positively related with CTC quantity in PDAC blood (R=0.628, p=0.029; R=0.776, p=0.003, respectively) using IgG chips. Furthermore, serum LAMC2 levels offered significant improvement over using CA19-9 alone for the discrimination of PDAC. Joint serum LAMC2 and CA19-9 levels increased the net benefit proportion in early stage/operational PDAC patients. Using integrated profiling, we identified LAMA3 and LAMC2 as potential therapeutic targets and prognostic markers for PDAC, for which further validation is warranted.

## INTRODUCTION

Pancreatic cancer is the ninth most common cancer in the United States, with an average of 43,090 deaths from pancreatic cancer reported every 5 years, ranking it as the fourth leading cause of cancer-related death [1]. There is also a continuous increase in pancreatic cancer incidence and mortality in China. As per current statistics, it is the seventh most common cancer diagnosed in men and the fourteenth in women, and the sixth leading cause of cancer deaths in men and eighth in women, with 65,600 new cases of pancreatic cancer (39,200 for men and 26,400 for women) and 63,500 deaths (26,400 for men and 25,800 for women) being reported in 2012 [2]. The relative 5-year survival rate is merely 6% due to its aggressive tumor biology that is accompanied by extensive local and early metastatic spread [3]. The most common histological type of pancreatic cancer is pancreatic ductal adenocarcinoma (PDAC), which accounts for most human pancreatic cancer cases (>95%) [4]. The most commonly used tumor marker, carbohydrate antigen 19-9 (CA19-9), is not very accurate for PDAC detection [5, 6]. CA19-9 is not obviously elevated during the early stages of the disease but is elevated in other benign conditions and multiple cancer types [7, 8]. As the most commonly used biomarker for the diagnosis of pancreatic cancer, sensitivity and specificity of CA19-9 can be as low as 70% with a 5% error rate [7, 9]. Taken together, it is critical to identify other biomarkers that can improve or complement the sensitivity and specificity of CA19-9.

Laminins are a family of extracellular matrix glycoproteins that are the major non-collagenous constituent in basement membranes. They have been implicated in a wide variety of biological processes including cell adhesion, differentiation, migration, signaling, neurite outgrowth and metastasis [10]. At present, human beings are known to have five α, four β, and three γ chains, encoding by LAMA1, LAMA2, LAMA3, LAMA4, LAMA5; LAMB1, LAMB2, LAMB3, LAMB4; and LAMC1, LAMC2 and LAMC3, respectively [11, 12]. Many studies have reported that the abnormal expression of genes of this family is associated with biological characteristics and clinical outcomes of cancers, such as gastric cancer [13], hepatocellular carcinoma [14], renal cell carcinoma [15], colorectal cancer [16] and lung cancer [17]. However, it remains unknown whether the key subunits of the laminin gene family are able to act as diagnostic, prognostic or therapeutic biomarkers for PDAC patients.

In the present study, we analyzed the expression and prognostic value of genes of laminin family in PDAC tissues using public online databases. In order to investigate potential pathways and related molecular mechanisms, we further performed enrichment and interaction network analysis. Moreover, the significance of the expression of key subunits in PDAC blood cells and circulating tumor cells (CTCs) and their serum levels were analyzed.

## RESULTS

### Initial screening of differential genes in pancreatic cancer and survival analysis

**Figure 1:**
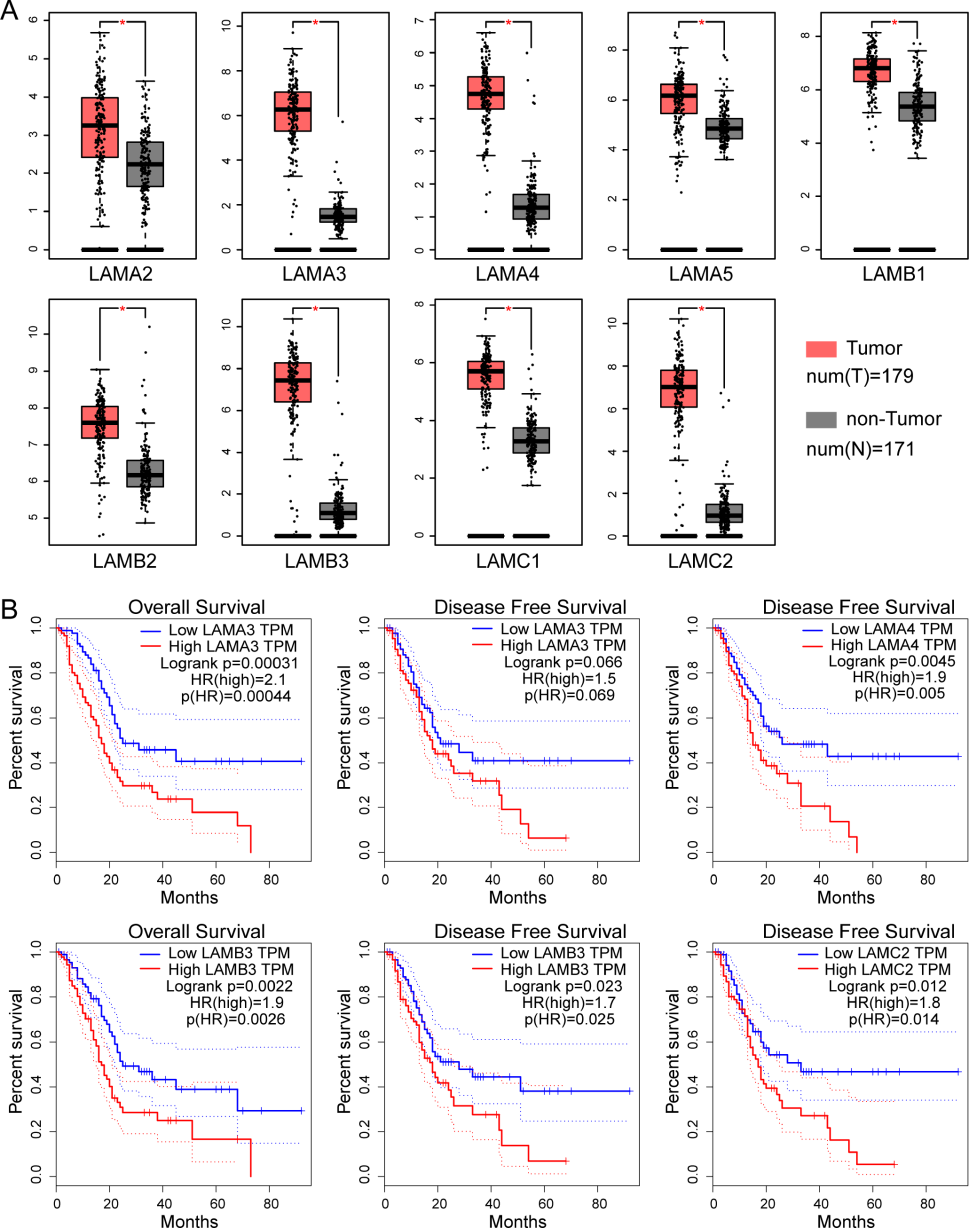
**Expression and survival analysis of the laminin gene family in TCGA PAAD cohort.** (**A**) Differential expression of genes of the laminin family in pancreatic cancer and non-tumor tissues. The red asterisk indicates that the difference is statistically significant (p<0.05). (**B**) Kaplan–Meier survival curves of the OS and DFS for high and low laminin gene expression groups, depicted using GEPIA. The cutoff value was set at median expression. The dotted line indicates a 95% confidence interval.

The differential expression of genes of laminin family in The Cancer Genome Atlas (TCGA) pancreatic adenocarcinoma (PAAD) cohort tumor and non-tumor tissues analyzed by Gene Expression Profiling Interactive Analysis (GEPIA, http://gepia.cancer-pku.cn/). All parameters were set to default values and patients were divided into two groups based on median expression. The results show that 9 laminin subunit genes (LAMA2, LAMA3, LAMA4, LAMA5, LAMB1, LAMB2, LAMB3, LAMC1, LAMC2) were highly expressed in tumor tissues of PAAD, with differences that were statistically significant (Figure 1A). In the survival analysis, high LAMA3 and LAMB3 expression groups were found to be related to adverse OS outcome in the PAAD cohort (log-rank p=0.0031 and 0.0022, respectively; Figure 1B). Additionally, high LAMA4, LAMB3 and LAMC2 expression groups were found to have a higher risk with regard to DFS in PAAD patients (HR=1.9, 1.7 and 1.8, respectively; Figure 1B).

### Kyoto encyclopedia of genes and genomes (KEGG) pathway, gene ontology (GO) term and interaction network analysis of genes of laminin family

**Figure 2:**
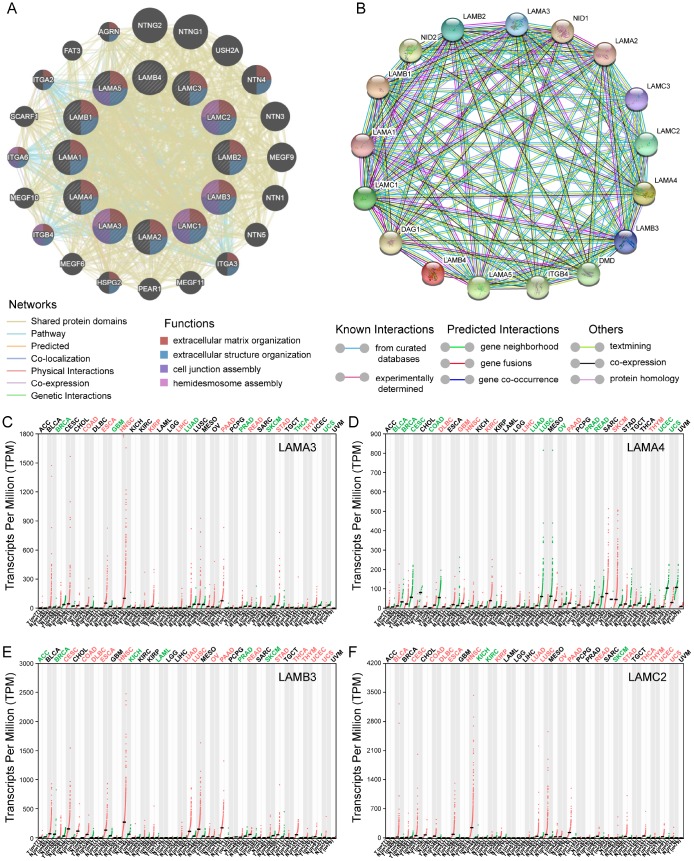
**Gene-gene interaction and protein-protein interaction network of genes of the laminin family, and differential expression of laminin genes in various tumor and non-tumor tissues.** (**A**) The gene network associated with the laminin gene family, drawn using GeneMANIA. The colored patches on the circle indicate the function of the gene. (**B**) A network diagram of interactions between proteins encoded by genes of the laminin family, drawn using STRING. (**C**–**F**) The difference in expression of LAMA3, LAMA4, LAMB3 and LAMC2 in various tissues in TCGA, drawn using GEPIA. Green color indicates that the gene is downregulated, whereas red color indicates upregulation of the gene.

The KEGG pathway and GO term analyses were performed using the *Clusterprofiler* R package (Supplementary Figure 1). The results demonstrated that products encoded by the genes of laminin family may be involved in the formation of extracellular matrices and are associated with cellular focal adhesion. Furthermore, genes of this family are involved in the PI3K-Akt signaling pathway and were found to partially bind to integrin subunits (ITG) in the enrichment analysis (Supplementary Figure 1). The gene-gene interaction network generated using GeneMANIA showed that genes in the laminin family may be associated with ITG and genes of netrin family (Figure 2A). In the protein-protein interaction network analysis conducted using the Search Tool for the Retrieval of Interacting Genes/Proteins (STRING), the laminin proteins were found to have a co-expression relationship with ITG and nidogen proteins (Figure 2B).

### Profiling of the expression in TCGA of various cancers and survival analysis using the gene expression omnibus (GEO) database for further selection

**Figure 3:**
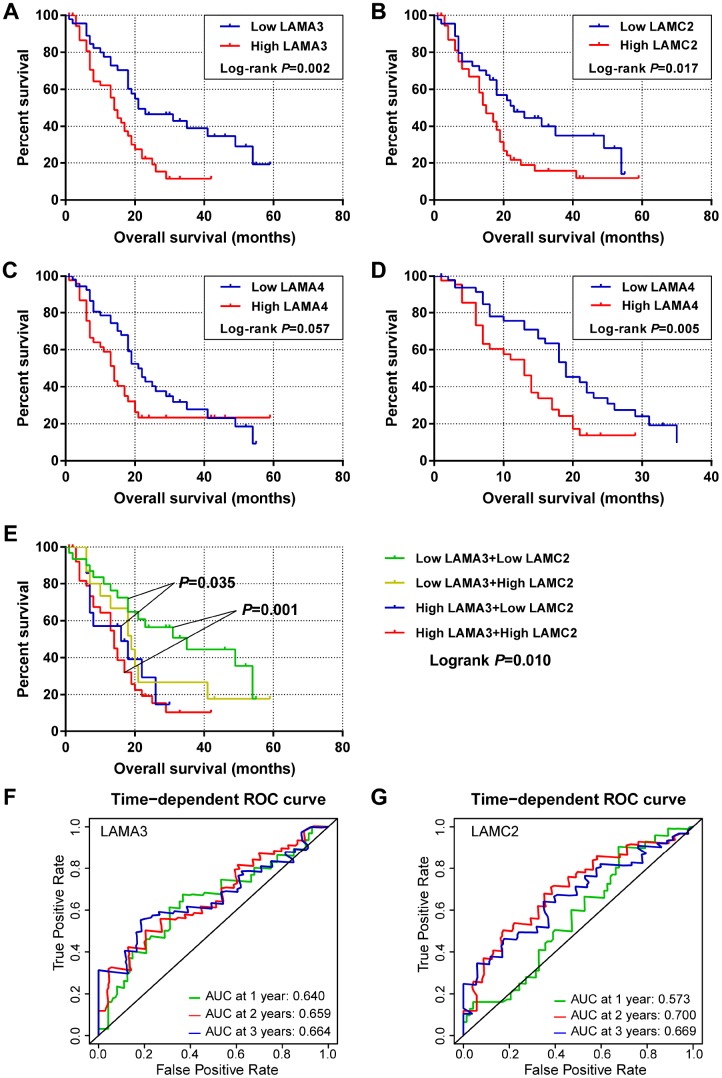
**Survival and survivalROC analysis of LAMA3 and LAMC2 expression in the GSE21501 PDAC cohort.** (**A**–**C**) Kaplan–Meier survival curves for OS of the LAMA3, LAMC2 and LAMA4 gene expression groups. (**D**) Kaplan–Meier survival curves for 3-year OS for LAMA4 expression groups. (**E**) Kaplan–Meier survival curves for OS of the combined LAMA3 and LAMC2 gene expression groups. (**F**–**G**) Time-dependent ROC curve for LAMA3 and LAMC2 expression in PDAC patients.

For the laminin subunits (LAMA3, LAMA4, LAMB3 and LAMC2) that were found to be associated with the prognosis of pancreatic cancer, we further analyzed their TCGA tumor tissue expression profiles (Figure 2C–2F). The results show that the expression of these genes may be upregulated in some of digestive tract tumors. Using the GSE21501 PDAC cohort, we further studied the relationship between the expression of genes of laminin family and prognosis of PDAC patients. Setting the median as the cutoff value, the Kaplan–Meier survival curves indicated that LAMA3 and LAMC2 expression are associated with the OS of PDAC patients (log-rank p=0.002 and 0.017, respectively; Figure 3A–3B). Although the survival analysis showed that LAMA4 gene expression and OS of PDAC did not reach statistical significance, it was also found to be associated with the 3-year OS of PDAC (Figure 3C–3D). However, no statistically significance was found in the association between LAMB3 expression and the OS of PDAC patients (Supplementary Table 1). The combined survival analysis demonstrated that the combination of high LAMA3 expression along with high or low expression of LAMC2 can have an adverse effect on the OS of PDAC patients (Figure 3E, Supplementary Table 2). The area under the curve (AUC) of the time-dependent receiver operating characteristic (ROC) curve for LAMA3 and LAMC2 indicating 1-, 2- and 3-year OS fluctuated between 0.6 and 0.7 (Figure 3F–3G).

### Tissue expression profiling and diagnostic efficacy evaluation of LAMA3 and LAMC2 in PDAC

**Figure 4:**
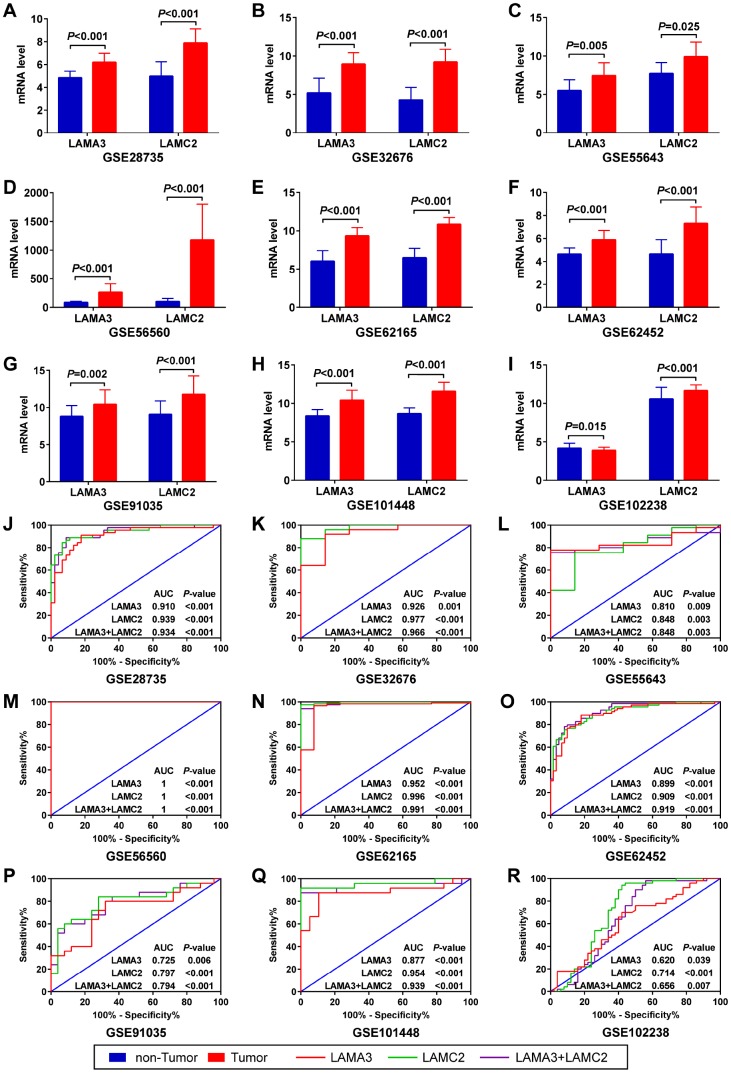
**Expression and ROC analysis of LAMA3 and LAMC2 in GEO PDAC and non-tumor tissues.** (**A**–**I**) Comparison of LAMA3 and LAMC2 gene expression in tumor and non-tumor tissues. (**J**–**R**) ROC curve of LAMA3, LAMC2 and combined expression for distinguishing between PDAC and non-tumor tissues.

**Figure 5:**
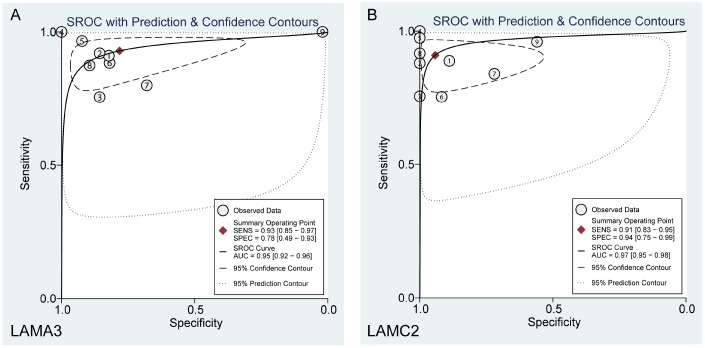
Summary ROC curve of (**A**) LAMA3 and (**B**) LAMC2 in GEO PDAC patients.

After filtering using the previous steps, we explored the expression of LAMA3 and LAMC2 in Genotype-Tissue Expression (GTEx) normal tissues and found that the expression of LAMA3 and LAMC2 were downregulated in pancreas tissues (Supplementary Figure 2). Using a selection process to identify tumor histology diagnosis of PDAC sets in GEO, 9 sets were included in this study (Supplementary Table 3). The results show that LAMA3 and LAMC2 expression is significantly upregulated in PDAC tumor tissues in most studies (Figure 4A–4I). The expression of LAMA3, LAMA4, LAMB3 and LAMC2 in each sample of the 9 sets, and the expression of all laminin genes in GTEx normal tissues are shown in the heatmap (Supplementary Figure 3 and Figure 4A, respectively). The ROC analysis of LAMA3, LAMC2 and their combined expression in PDAC sets indicate that both genes display high accuracy in distinguishing between tumor and non-tumor tissues (the AUCs of the ROC curves were >0.70, Figure 4J–4R). The summary receiver operating characteristic (SROC) curves indicate that the AUCs of LAMA3 and LAMC2 are more than 0.90 in PDAC patients (Figure 5).

### Joint diagnostic value and benefit comparison of serum LAMC2 and CA19-9 levels

**Figure 6:**
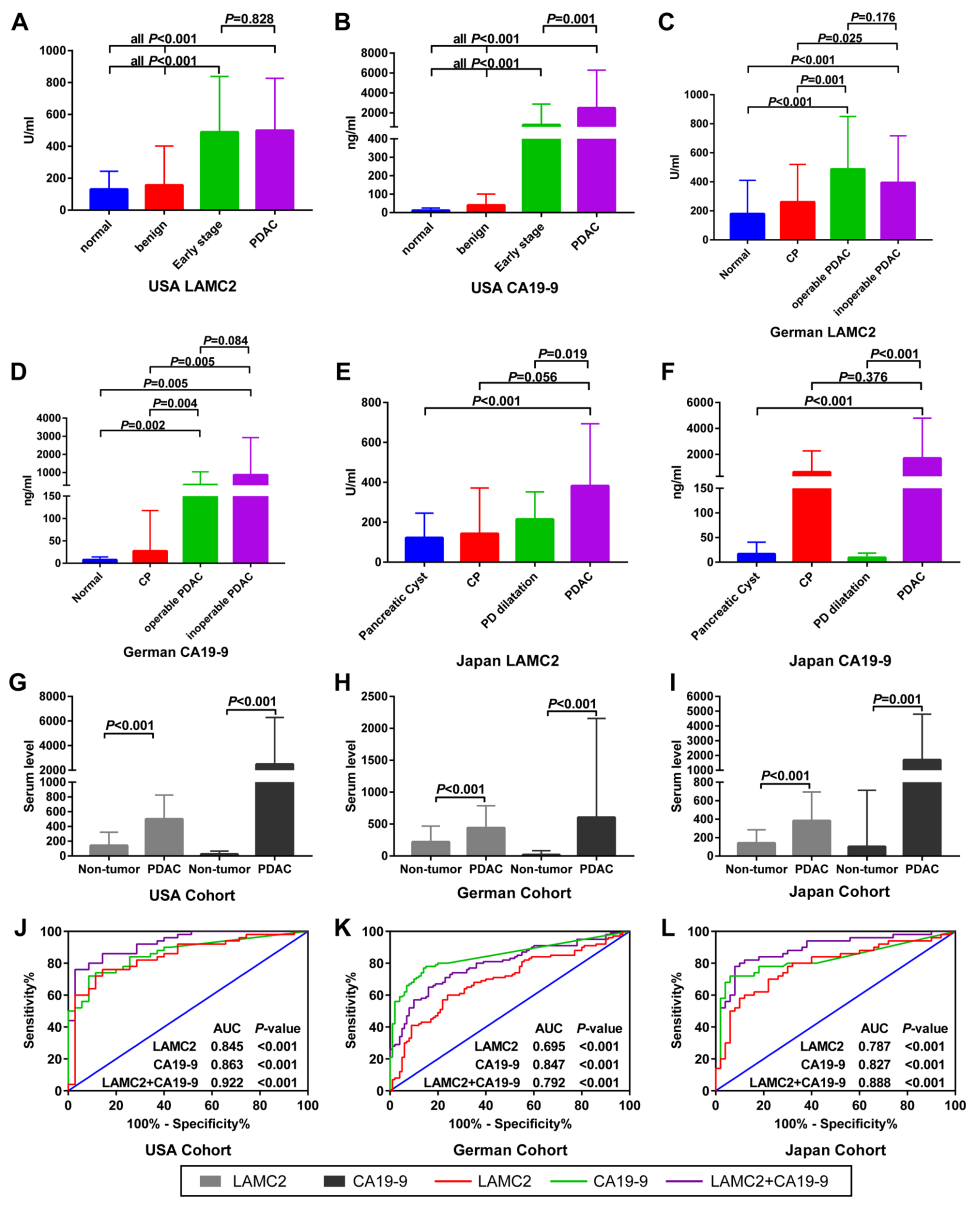
**Serum levels and ROC analysis of LAMC2 and CA19-9 expression in PDAC and non-tumor patients.** (**A**–**F**) Comparison of LAMC2 and CA19-9 serum levels in PDAC and non-tumor patients from the USA, German and Japan cohorts, respectively. (**G**–**I**) Comparison of LAMC2 and CA19-9 serum levels in PDAC and non-tumor patients combined from all cohorts. (**J**–**L**) ROC curve of LAMC2, CA19-9 and combined serum levels in distinguishing between PDAC and non-tumor tissues from USA, German and Japan cohorts, respectively. CP, Chronic pancreatitis; PD, Pancreatic duct.

**Figure 7:**
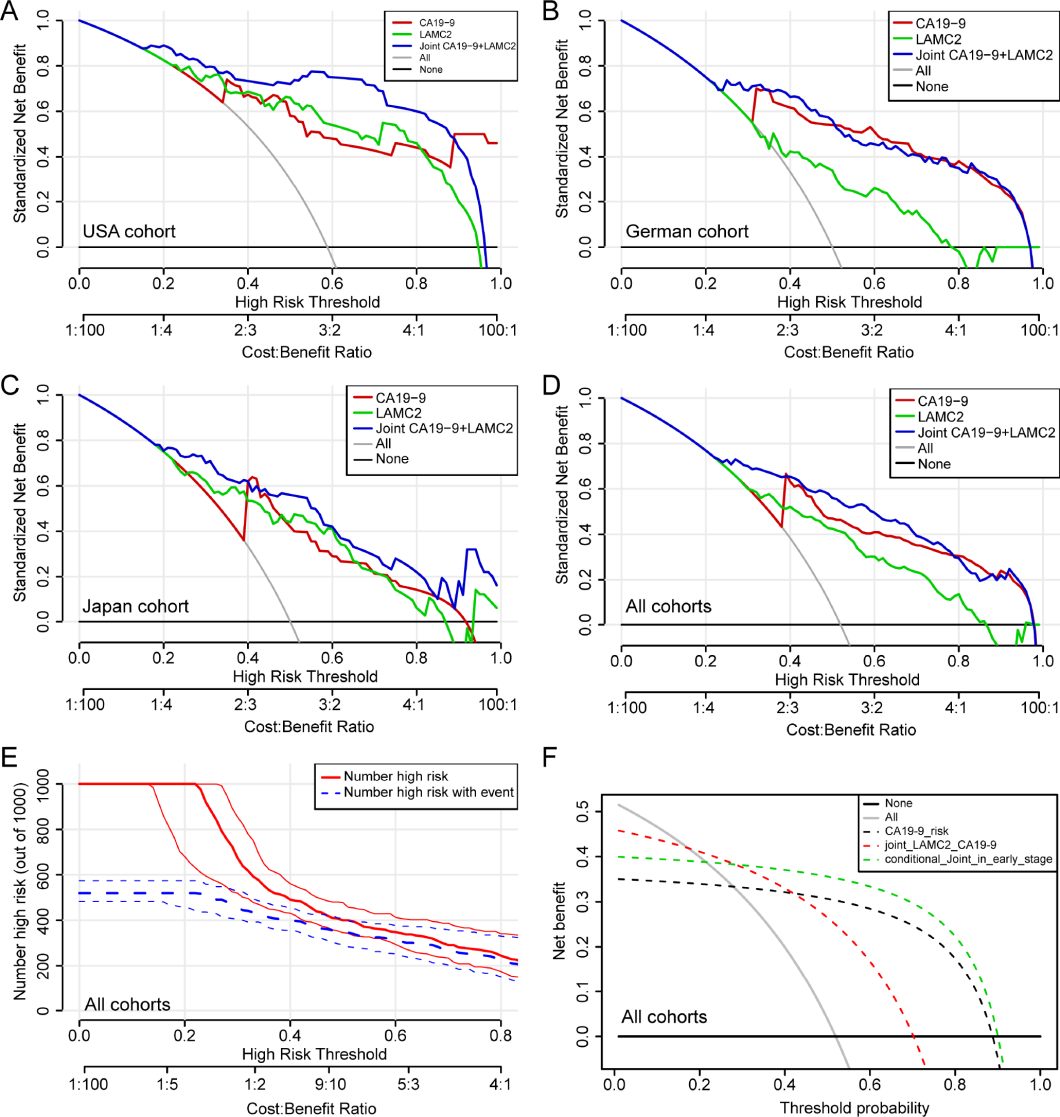
**Decision curve analysis for the serum levels of LAMC2 and CA19-9 in PDAC patients.** (**A**–**D**) Net benefit of LAMC2 and CA19-9 threshold probability from USA, German, Japan and combined cohorts, respectively. (**E**) Net benefit and Bootstrapping with PDAC high risk number resamples per 1000 patients of LAMC2 and CA19-9 threshold probability from the combined cohort. The red line indicates the number of people who are classified as positive (high risk) by the joint serum model under each threshold probability; the blue line (the number of high risk with event) is the number of true positives under each threshold probability. (**F**) Joint and conditional decision curve analysis of LAMC2 and CA19-9 threshold probability of all cohorts. The red dotted line represents the joint high serum levels of LAMC2 and CA19-9. The green dotted line represents joint high serum levels of LAMC2 and CA19-9 in early stage and operable PDAC patients.

Compared with normal or benign pancreatic tissues, the serum levels of LAMC2 and CA19-9 have been found to be elevated in PDAC patients from USA, German and Japan cohorts, with different groups reaching varied levels of statistical significance (p<0.05, Figure 6A–6F). Results of the combined analysis suggest that serum level of LAMC2 and CA19-9 were higher in the PDAC patients than that of non-tumor patients/heathy donor from USA, German and Japan cohorts (p<0.05, Figure 6G–6I, Supplementary Table 3). Furthermore, serum LAMC2 levels can be used to significantly improve the diagnostic accuracy of using CA19-9 for PDAC patients, since the AUC for distinguishing between tumor and non-tumor tissues for the combined analysis of LAMC2 and CA19-9 (AUC=0.922, 0.792 and 0.888, respectively) in two of three cohorts were higher than the AUC values obtained for CA19-9 alone (AUC=0.863, 0.847 and 0.827, respectively; Figure 6J–6L). In the decision curve analysis, we found that the proportion of standardized net benefit populations increase rapidly for serum CA19-9 when the threshold probability is between 0.3-0.4 (Figure 7A–7D). Net benefit populations improved in the joint LAMC2 and CA19-9 analysis at a low threshold probability, indicating that there is diagnostic complementarity between LAMC2 and CA19-9 (cost and benefit ratio is significantly reduced) (Figure 7A–7D). The standardized net benefit proportion of every 1000 resamples had a threshold probability of less than 0.3 and was higher than other thresholds (Figure 7E). Moreover, joint use for the diagnosis of significantly increased the percentage of the early stage/operable PDAC benefiting population, suggesting that the clinician can diagnose PDAC in the patient group earlier (Figure 7F).

### Blood cell and CTC expression profiling of LAMA3 and LAMC2 and CTC number evaluation in PDAC patients

**Figure 8:**
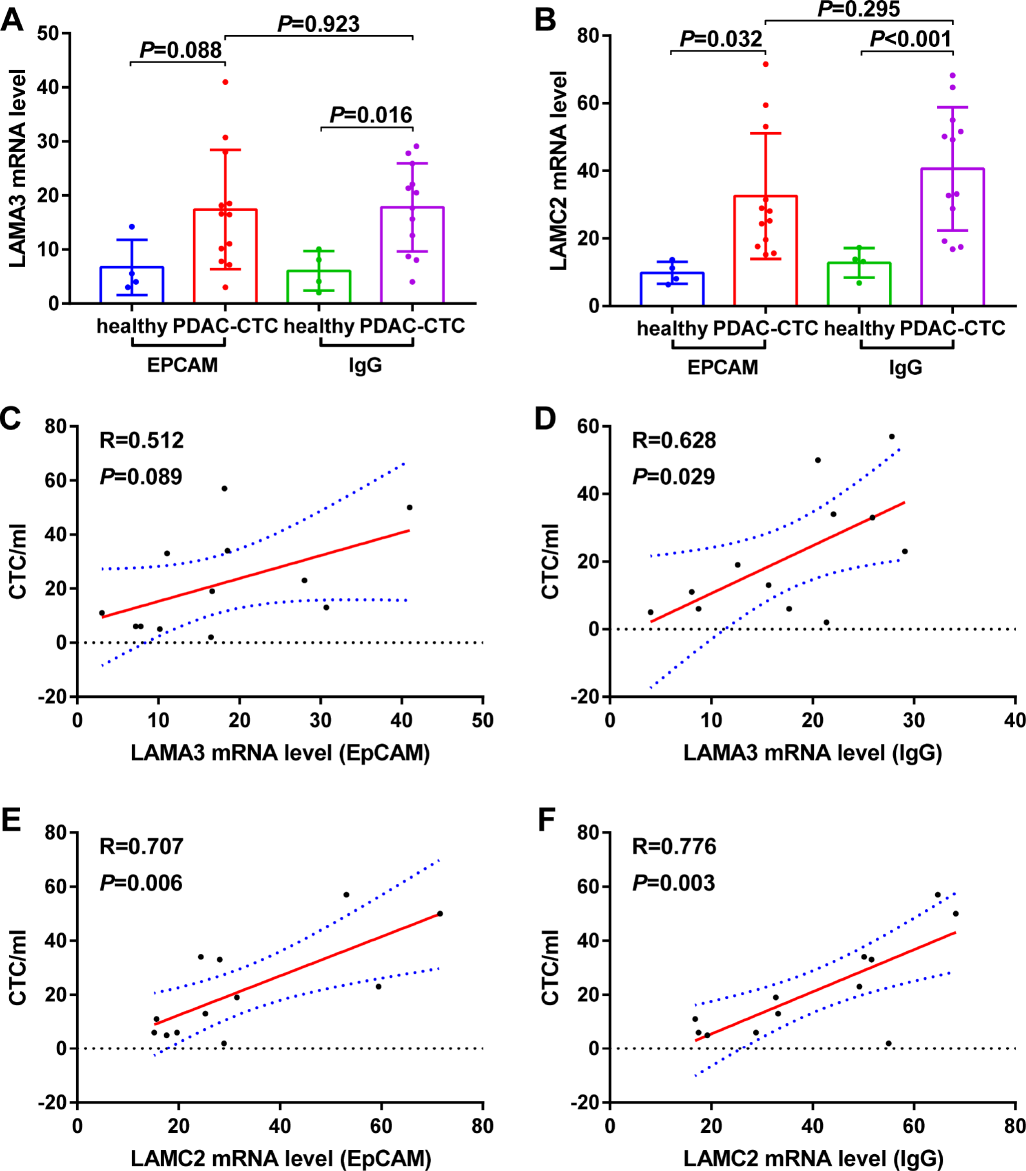
Expression levels of (**A**) LAMA3 and (**B**) LAMC2 in heathy donor and PDAC Circulating tumor cells (CTCs) and the relationship between (**C**–**D**) LAMA3 and (**E**–**F**) LAMC2 expression and CTC number in PDAC patients as processed using EpCAM and IgG CTC–Chip.

The expression of LAMA3 and LAMC2 in PDAC CTC were higher than that of healthy donor whole blood cells when the analysis was performed using GSE40171 expression profiling (Figure 8A–8B). Furthermore, the correlation analysis indicates that high LAMA3 expression is positively related with the number of CTC in the blood using IgG chips (R=0.628, p=0.029, Figure 8D) but not using EpCAM chips (R=0.512, p=0.089, Figure 8C). However, high LAMC2 expression was found to be statistically positively related with the number of CTC in the blood using both EpCAM (R=0.707, p=0.006, Figure 8E) and IgG chips (R=0.776, p=0.003, Figure 8F). Expression of LAMA3 and LAMC2 were found to be at low levels in whole blood cells of the GTEx heathy cohort (Supplementary Figure 2). There was no statistical difference between the expression of LAMA3 and LAMC2 from PDAC patients in peripheral mononuclear cells and whole blood cells compared with that of heathy controls (Supplementary Figure 4B–4C).

## DISCUSSION

In the present study, we initially screened out four differentially expressed subunits (LAMA3, LAMA4, LAMB3 and LAMC2) of the laminin gene family that were found to be associated with the clinical outcome of pancreas cancer patients in TCGA. Using external verification for GEO PDAC datasets, the two key subunits (LAMA3 and LAMC2) that were found to be associated with the OS of PDAC patients were further analyzed. The expression of LAMA3 and LAMC2 was found to be upregulated in PDAC tumor tissues in the 9 datasets, with a favorable diagnostic ability for distinguishing between PDAC and non-tumor tissues. Additionally, high levels of LAMA3 and LAMC2 expression were found in PDAC CTCs and these expression levels were found to be positively related with CTC number in the blood of PDAC patients. Furthermore, we identified that serum LAMC2 levels present a high diagnostical ability and significant improvement over that of CA19-9 alone for the discrimination of PDAC tissue from that of healthy controls and benign subjects. Using joint application of serum LAMC2 and CA19-9, it was found that the proportion of net benefit populations increased, particularly among early stage/operational PDAC patients.

LAMA3 and LAMC2 encode for products that are essential parts of laminin-322 (formerly known as laminin-5), which is known to stimulate cell migration in cancers [18, 19] and is related with the prognosis of diseases [20]. In this study, results of the enrichment analysis and interaction networks indicate that laminin subunits are closely related with ITG. The ITG family of proteins are essential focal adhesion proteins that are found in various cancer cell types, and may affect the initiation, proliferation, survival and metastasis of tumors [21], and have been found to be correlated with the poor differentiation status and metastatic potential of PDAC [22]. The relationship between cell adhesion of laminins and ITGs in pancreatic carcinoma cells has been investigated. Rosewicz et al. found that retinoids decrease pancreatic carcinoma cell adhesion to the laminin via alteration of the integrin receptor function and thereby modulate infiltrative growth and metastasis of pancreatic cancer [23], but the specific mechanism needs to be identified.

Our results demonstrate that the expression of LAMA3 and LAMC2 is related to the prognosis of PDAC, is highly elevated in CTCs and positively correlated with the number of CTCs in blood. Previous research [24] has indicated that LAMC2 fragment circulating levels are markedly elevated in PDAC patients with liver metastases, which is independent of basement membrane metabolism in the entire body. In addition, a study on gastric cancer [25] has suggested LAMB3 and LAMC2 chains accumulate intracellularly and are related with cancer progression, while epigenetic silencing of the LAMA3 chain may lead to an inability of synthesizing the basement membrane, which may affect cancer cell invasion. These results suggest that there may be some differences in the biological function of LAMA3 and LAMC2 regarding tumor progression.

In the expression level difference analysis, LAMA3 mRNA expression in PDAC non-tumor tissues in GSE102238 was lower when compared with tumor tissues from paired patients whose histopathology confirmed various extents of neurological invasion. However, LAMC2 expression was still elevated in PDAC tumor tissues. Mitsunaga *et al*. found that LAMC2 plays an important role in nerve invasion, with high LAMC2 mRNA and protein expression being associated with long nerve invasion [26]. Further, they studied the knockdown of LAMC2 in cancer cells, which was found to significantly shorten nerve invasion in an animal model. Our results indicate that LAMC2 expression in GSE102238 tumor tissues is higher than that of paracancer tissues of PDAC and patients who harbored high levels of LAMC2 expression had a poor prognosis. We hypothesize that high expression of LAMC2 promotes PDAC cell invasion of the nerve and leads to an increased risk of tumor recurrence.

LAMC2 is composed of epidermal growth factor (EGF)-like repeats that allow it to interact with EGFR (Epidermal Growth Factor Receptor). This binding stimulates downstream signaling that results in increased MMP-2 gene expression, which can enhance cell migration in breast carcinoma [27]. Another study identified signaling events coordinated by EGF and a specific ITG that regulates the invasive behavior of carcinoma cells. EGF stimulation of pancreatic carcinoma cells led to invasion and metastasis that was blocked by antagonists of ITG [28]. Additionally, the elevated expression of LAMC2 in cancer cells appears to drive tumorigenesis, through its interactions with several cell-surface receptors, including ITGs and EGFRs [29]. These results suggest that the interaction between laminins and ITGs may play a role in tumor invasion and metastasis of the EGF pathway, but the specific mechanism needs further verification.

An immunohistochemical study reported that the expression of LAMA3 and LAMC2 may serve an important role in identification of the progression and prognosis of PDAC patients [30]. According to immunohistochemical expression patterns, the basement membrane type of LAMC2 is correlated with differentiation and low risk for the prognosis of PDAC. The cytoplasmic expression of LAMC2 represents the high invasive potential of the tumor and is correlated with distant metastasis, especially hepatic metastasis, and with a poorer prognosis in patients with PDAC [31]. The above results indicate that the expression patterns of LAMC2 have different biological behaviors in tumor cells.

In PDAC tumor tissues, the expression of LAMA3 and LAMC2 are upregulated with a favorable ability of distinguishing between PDAC and non-tumor tissues, which is of a prognostic value for PDAC patients. However, histological analysis is an invasive examination that is not suitable for the early diagnosis of PDAC, in terms of safety. Therefore, serum mRNA detection can provide a good alternative method to assist clinicians with diagnosis. Considering some of the challenges encountered regarding the sensitivity and specificity of CA19-9 in diagnosis, Makawita *et al*. [32] investigated additional serum biomarkers that show promising results as improved diagnostic indicators of pancreatic cancer when combined with CA19-9. Kanda *et al*. reported that the preoperative index, which was obtained by multiplying the values of CA19-9 and carcinoembryonic antigen (CEA), had a strong correlation with the prognosis of patients with pancreatic cancer [33].

Moreover, our results illustrate that LAMA3 and LAMC2 expression levels are strong prognostic biomarkers that could help identify PDAC patients expected to have a poor prognosis, in order that they can be administered appropriate multidisciplinary treatment. Since the original study did not analyze differences in serum LAMC2 and CA19-9 levels and overall discrimination between groups in the included population [34], we further analyzed the diagnostic capabilities of LAMC2 and CA19-9 alone and jointly at serum level in all research cohorts using supplemental material. The results of a recent study validated the ability of the joint application of serum levels of LAMC2 and CA19-9 for discriminating PDAC patients from benign patients in the training and validation cohorts [35]. The decision curve analysis in our study also indicates that the proportion of net benefit populations increased in early stage/operational PDAC patients using joint serum LAMC2 and CA19-9 levels. The cost-effectiveness ratio decreased in joint serum LAMC2 and CA19-9. Further, the researchers identified a biomarker panel consisting of CA19-9, CA125 and LAMC2, which was found to be better at detecting PDAC patients than CA19-9 alone, most notably during the early stages of the disease [35]. Based on these research results, the joint analysis was found to have certain advantages compared with the single factor analysis. The expression levels of laminin subunits did not rise in PDAC patients, compared with that of peripheral mononuclear cells and whole blood cells of heathy controls. Regrettably, levels of other serological laminins were not obtained in this study due to data relevance, and therefore the combined value of LAMA3 serum levels and that of CA19-9 could not be analyzed. The relationship among the level of serological laminins, CTCs in the blood, expression in tumor tissues and prognosis of PDAC patients need further research. Real-world clinical applications should be further validated based on multicenter early stage PDAC samples.

For clinical translational medicine, we obtained the diagnostic and prognostic value of genes in the laminin family for pancreatic cancer, which can serve the following clinical applications: 1) Detection of laminin gene family expression levels in tumor tissue to assist in distinguishing pancreatic cancer types and assessing the risk of metastatic recurrence; 2). Detection of laminin serological levels to improve the diagnostic ability of CA199 in pancreatic cancer and to estimate the number of circulating tumor cells to assess the risk of metastasis.

In summary, we screened two overexpressed key laminin subunits (LAMA3 and LAMC2) that facilitate the occurrence and progression of PDAC. The serum level of LAMC2 offers significant improvement over CA19-9 alone for the discrimination of PDAC tissue from that of healthy controls and benign subjects. Joint serum LAMC2 and CA19-9 levels can increase the net benefit proportion of early stage/operational PDAC patients. These findings provide a new perspective on the underlying molecular mechanism of laminin subunits in PDAC, suggesting that key laminin subunits may be valuable biomarkers and therapeutic targets for PDAC patients.

## MATERIALS AND METHODS

This study was approved by the Ethics Committee of the First Affiliated Hospital of Guangxi Medical University (Guangxi, China).

### Profiling of laminin gene family in TCGA and GTEx tissues

GEPIA (http://gepia.cancer-pku.cn/) [36] is a data visualization website for analyzing RNA-Seq expression data from TCGA and GTEx projects. The comparation of the expression of genes of the laminin gene family in PAAD and non-tumor tissues was performed using GEPIA. Then, the expression of genes of the laminin family were also analyzed in various tumor and non-tumor tissues using GEPIA. Profiling of the laminin gene family in normal tissues was visualized and data were extracted from the GTEx portal (https://www.gtexportal.org/).

### Bioinformatics analysis of genes of the laminin family

In order to investigate the biological functions and pathways of the genes of the laminin family, the *Clusterprofiler* [37] R package was used to perform KEGG pathway and GO term enrichment analyses. The gene-gene and protein-protein interaction networks of genes of the laminin family were explored using GeneMANIA (http://www.genemania.org/) [38] and STRING (https://string-db.org) [39, 40], respectively.

### Prognostic value of genes of the laminin family in PDAC patients

The association between genes of the laminin family and the clinical outcome of PAAD patients was analyzed using GEPIA. In order to validate the relationship between genes of the laminin family and the clinical outcome of PDAC patients, we looked for datasets containing data of histopathologically confirmed PDAC patients with complete clinical outcome data in GEO (http://www.ncbi.nlm.nih.gov/geo). Then, the GSE21501 [41] PDAC dataset was included in this study but cases with incomplete prognostic information were excluded. The median expression level was set as cutoff value for categorizing patients into high and low expression groups. In order to evaluate the predictive accuracy of genes of the laminin family for the clinical outcome of PDAC patients, the *survivalROC* [42] R package was performed to construct a time-dependent ROC curve.

### Profiling of genes of the laminin family in GEO PDAC datasets

The validation of the expression level of genes of the laminin family in PDAC tumor tissues was further explored in GEO. The inclusion criteria were as follows: (i) expression data on genes of the laminin family available; (ii) total sample size of each dataset in tumor and non-tumor groups exceeds 30; (iii) human subjects; and (iv) tumor histopathologically confirmed as PDAC. As a result, a total of 9 datasets were further analyzed, which included GSE28735 [43, 44], GSE32676 [45], GSE55643 [46], GSE56560 [47, 48], GSE62165 [49], GSE62452 [50], GSE91035, GSE101448 [51] and GSE102238. Moreover, in order to investigate the expression of genes of the laminin family between PDAC blood cells, CTCs and healthy control blood cells, 4 datasets, GSE40171 [52], GSE49641 [53], GSE60601 and GSE76429, were included and supplementary data were collected.

### Serum levels of LAMC2 and CA19-9 in PDAC patients

By searching for supplementary data from published studies, we obtained serum data of LAMC2 and CA19-9 levels of three cohorts (USA, German and Japan) from a study conducted by Doctor Hari Kosanam from the University of Toronto [34]. Serum levels of LAMC2 and CA19-9 were detected using Enzyme-linked immunosorbent assays (purchased from USCN Life Sciences), according to the manufacturer’s protocols. Serum LAMC2 and CA19-9 levels between groups and the ability of joint serum levels in discriminating PDAC from non-tumor controls were further investigated. According to the original research reported [34], the cutoff values for LAMC2 and CA19-9 were set to 150 U/ml and 37 ng/ml, respectively. In order to evaluate the net benefit proportion, decision curve analysis [54] were constructed for serum LAMC2, CA19-9 and joint levels in different PDAC cohorts or stages. The decision curve, a graphical summary for assessing the potential clinical impact of risk prediction biomarkers or risk models for recommending treatment or intervention, allows one to examine a risk model performance across a range of plausible risk thresholds [55]. Equivalently, the decision curves allow for examination of risk models across a range of plausible cost-benefit ratios [56].

### Statistical analysis

All statistical analyses were performed using SPSS version 24.0 (IBM Corp., Chicago, IL, USA) and STATA version 13.0 (Stata Corp., College Station, TX, USA). A two-sided p value of <0.05 was considered to be statistically significant. Continuous variables with a normal distribution are presented as mean and standard deviation (SD) and the mean of two continuous normally distributed variables are compared using the independent samples Student’s test. The survival curves and heatmaps were constructed using GraphPad Prism 7.01 (GraphPad Software, Inc., San Diego, CA, USA). The Kaplan-Meier survival curves were compared using the log-rank test. Enrichment plot, time-dependent ROC curve and decision curve analysis were performed in R 3.4.1 (http://www.R-project.org/). The ROC curve was drawn to identify the diagnostic significance of the laminin gene family in the GEO dataset. The AUC value for assessing the predictive accuracy and discriminative ability of ROC were calculated using SPSS 24.0. A SROC curve and the AUC value of SROC were calculated using STATA 13.0. The relationship between laminin genes and CTC number was assessed using the Pearson’s correlation coefficient.

## Supplementary Material

Supplementary Figures

Supplementary Tables
