# Supplementary material for: Evaluation of the diagnostic ability of laminin gene family for pancreatic ductal adenocarcinoma
Source: Aging (Albany NY). 2019 Jun 10;11(11):3679–703. doi: 10.18632/aging.102007 (PMC6594799; doi:10.18632/aging.102007)
Supplement: Supplementary Figures [file aging-11-102007-s002.pdf]

SUPPLEMENTARY MATERIALS

Supplementary Figures

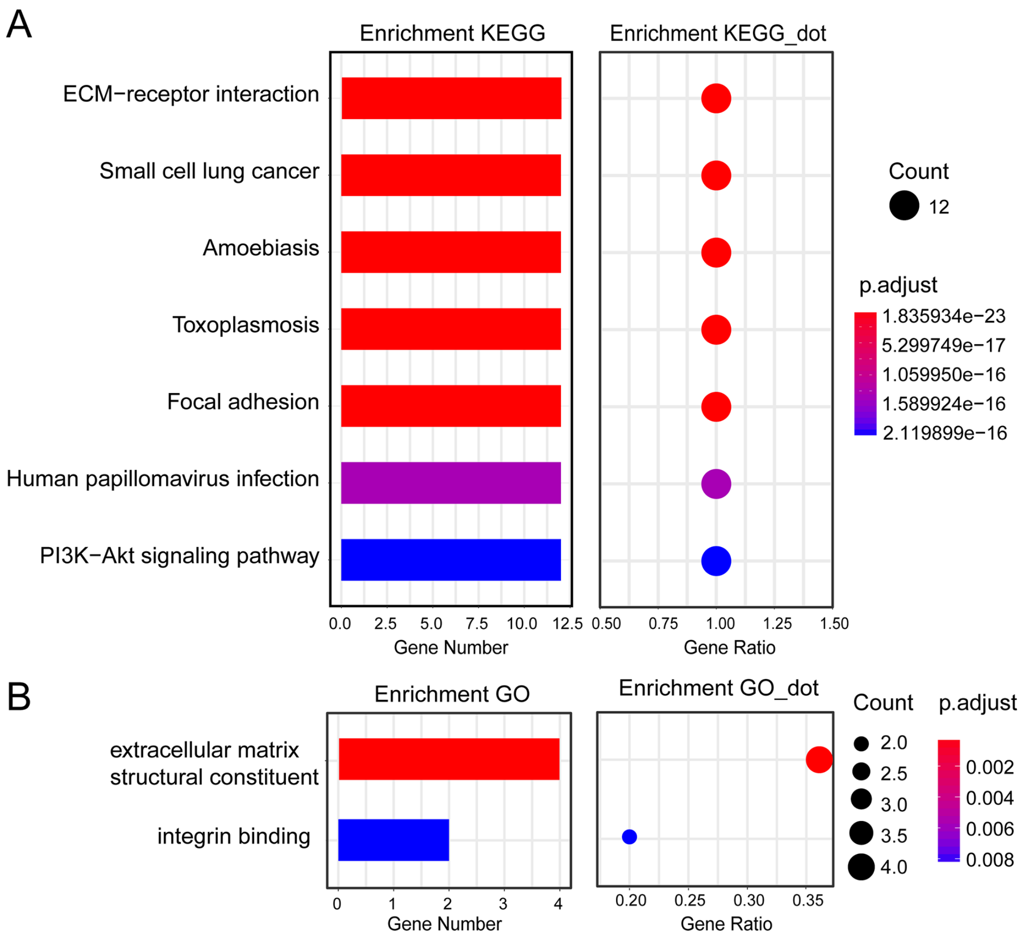

Supplementary Figure 1. (**A**) KEGG pathway and (**B**) GO term enrichment plots of genes of the laminin family enriched obtained through the *Clusterprofiler* R package**.**

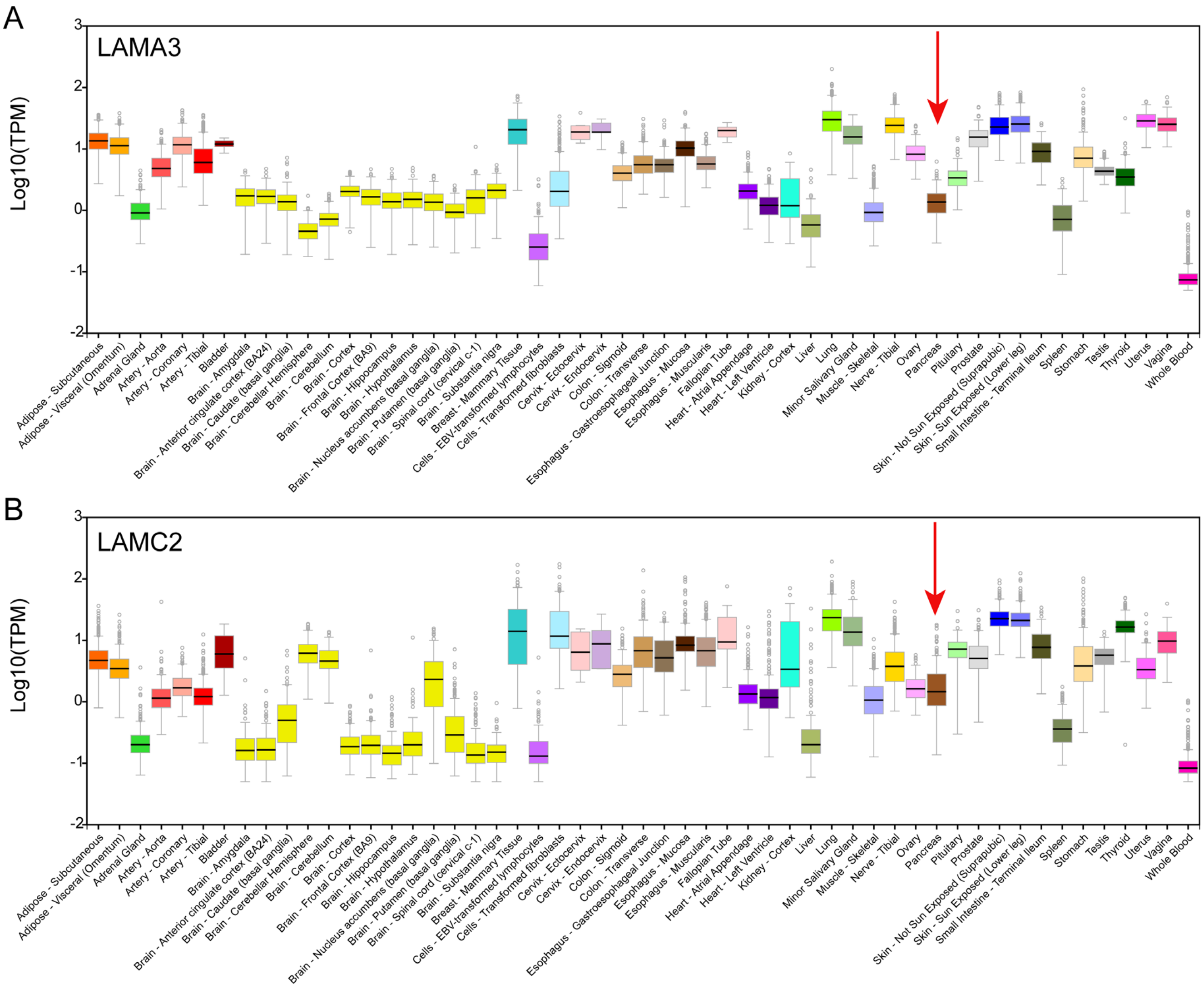

Supplementary Figure 2. Expression levels of (**A**) LAMA3 and (**B**) LAMC2 in various normal tissues as analyzed through the GTEx database. The red arrows indicate pancreatic tissue.

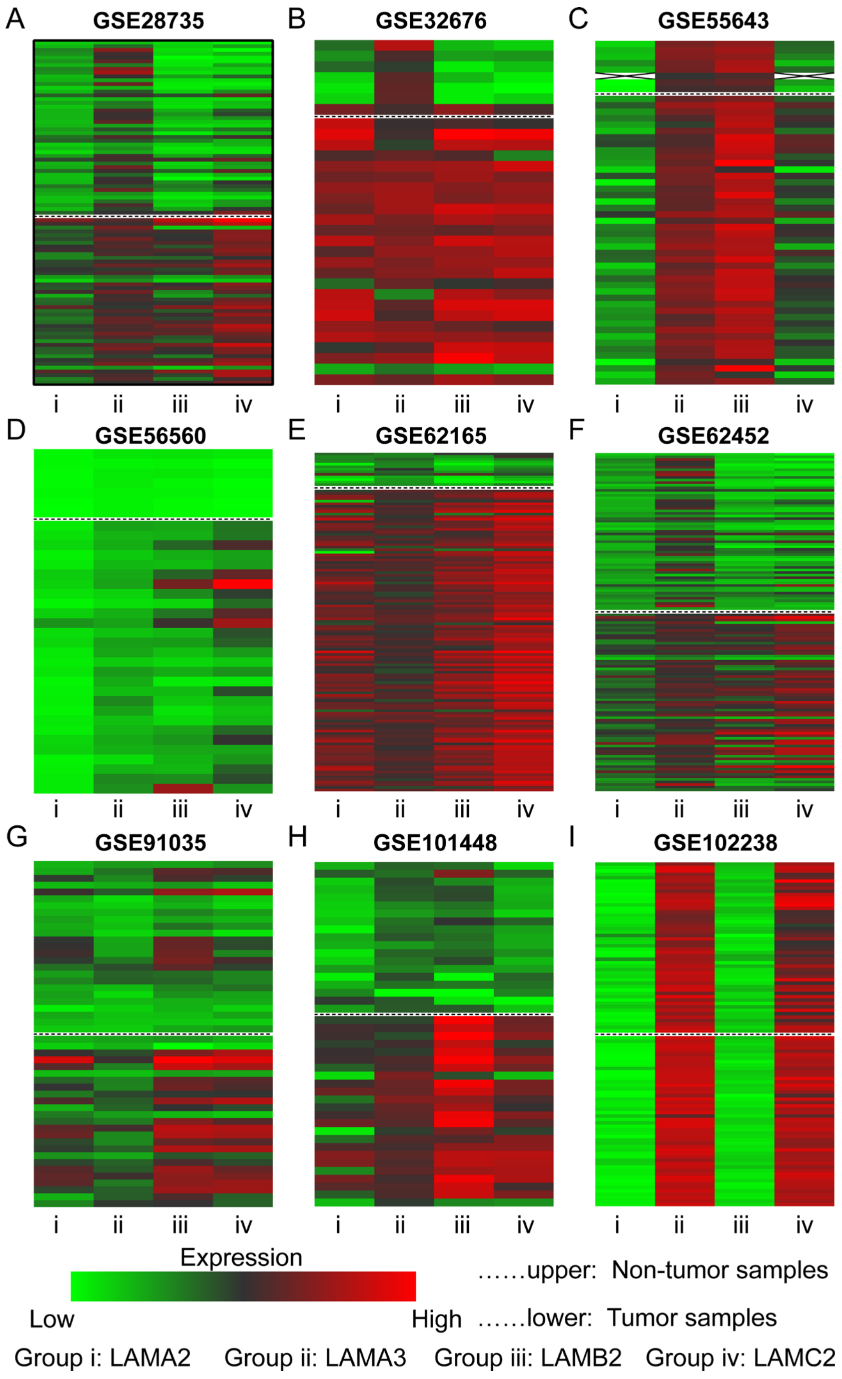

Supplementary Figure 3. Heatmap for LAMA3, LAMA4, LAMB3 and LAMC2 expression levels in GEO PDAC and non-tumor tissues. Green and red colors represent low and high mRNA expression levels, respectively. Group i-iv represent LAMA3, LAMA4, LAMB3 and LAMC2, respectively.

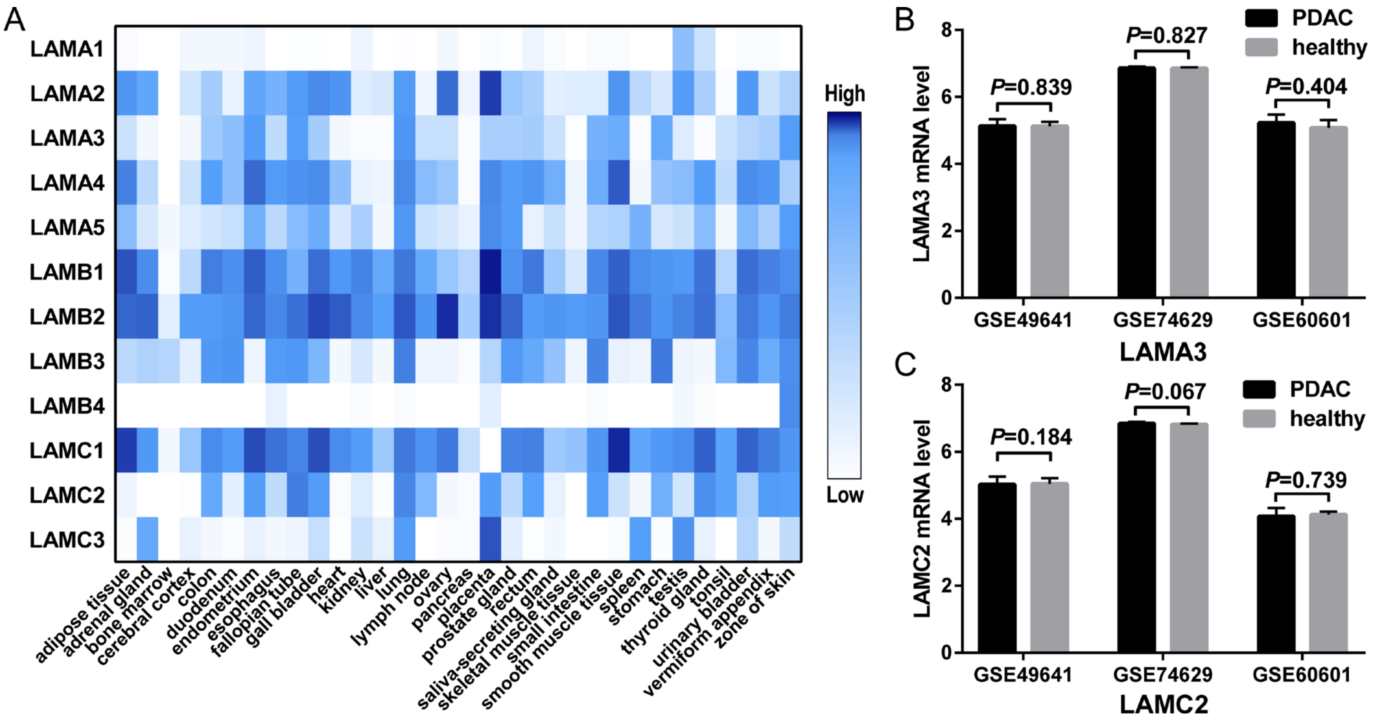

Supplementary Figure 4. (**A**) Expression of genes of the laminin family in GTEx normal tissues. (**B**–**C**) Expression of LAMA3 and LAMC2 between PDAC patient and healthy control blood samples (GSE49641: peripheral blood mononuclear cells; GSE74629: peripheral blood; GSE60601: classical CD14++ CD16- monocytes).
