# Supplementary material for: Evaluation of the diagnostic ability of laminin gene family for pancreatic ductal adenocarcinoma
Source: Aging (Albany NY). 2019 Jun 10;11(11):3679–703. doi: 10.18632/aging.102007 (PMC6594799; doi:10.18632/aging.102007)
Supplement: Supplementary Tables [file aging-11-102007-s001.pdf]

Supplementary Tables

Supplementary Table 1. Survival analysis of laminin gene family in GSE21501 PDAC patients.

Variables		MST (months)	*P* value	HR (95%CI)*	*P* value
LAMA1	low	19	0.425		0.435
	high	18		1.21(0.75-1.97)	
LAMA2	low	17	0.180		0.192
	high	19		0.72(0.44-1.18)	
LAMA3	low	21	**0.002**		**0.003**
	high	14		**2.18(1.30-3.65)**	
LAMA4	low	21	0.057		0.065
	high	14		1,59(0.97-2.59)	
LAMA5	low	21	0.051		0.059
	high	17		1.61(0.98-2.64)	
LAMB1	low	18	0.746		0.751
	high	19		1.08(0.66-1.77)	
LAMB2	low	18	0.439		0.449
	high	18		1.21(0.74-1.96)	
LAMB3	low	21	0.401		0.411
	high	17		1.23(0.75-2.00)	
LAMB4	low	19	0.557		0.566
	high	17		1.15(0.71-1.87)	
LAMC1	low	15	0.596		0.604
	high	19		0.88(0.54-1.43)	
LAMC2	low	23	**0.017**		**0.021**
	high	15		**1.80(1.09-2.97)**	
LAMC3	low	15	0.264		0.276
	high	21		0.76(0.47-1.24)	
LAMA4-3y	low	19	**0.005**		**0.007**
	high	13		**2.04 (1.21-3.42)**	

Notes: *HR for the univariate Cox proportional hazards regression analysis.
Abbreviations: PDAC, pancreatic ductal adenocarcinoma; MST, median survival time; HR, hazard ratio; CI, confidence interval.

Supplementary Table 2. Combined survival analysis of LAMA3 and LAMC2 expression in GSE21501 PDAC patients.

Variables	MST (months)	*P* value	HR (95%CI)	*P* value
Low LAMA3+Low LAMC2	35	**0.010**		**0.017**
Low LAMA3+High LAMC2	19		1.66(0.77-3.58)	0.201
High LAMA3+Low LAMC2	16		2.23(0.99-5.01)	0.053
High LAMA3+High LAMC2	14		2.77(1.47-5.24)	**0.002**

Notes: *HR for the univariate Cox proportional hazards regression analysis.
Abbreviations: PDAC, pancreatic ductal adenocarcinoma; MST, median survival time; HR, hazard ratio; CI, confidence interval.

Supplementary Table 3. Expression of laminin gene family in PDAC datasets and serum LAMC2 and CA19-9 level enrolled in this study.

Datasets	First author	Year	Variables	No.	non-tumor		No.	PDAC		*P* value	AUC	95%CI		*P* value
					Mean	SD		Mean	SD			lower	upper	
GSE28735 (1, 2)	Zhang G	2012	LAMA3	45	4.84	0.58	45	6.19	0.80	<0.001	0.910	0.847	0.972	<0.001
			LAMA4	45	6.42	1.17	45	7.18	0.66	<0.001	0.939	0.892	0.987	<0.001
			LAMB3	45	4.39	0.88	45	6.74	1.14	<0.001	0.700	0.590	0.811	0.001
			LAMC2	45	4.98	1.25	45	7.88	1.24	<0.001	0.937	0.890	0.985	<0.001
GSE32676 (3)	Donahue TR	2011	LAMA3	7	5.20	1.92	25	8.94	1.50	<0.001	0.926	0.820	1	0.001
			LAMA4	7	8.13	0.98	25	8.41	1.15	0.572	0.646	0.405	0.887	0.245
			LAMB3	7	4.52	2.16	25	9.39	1.50	<0.001	0.954	0.864	1	<0.001
			LAMC2	7	4.27	1.65	25	9.20	1.68	<0.001	0.977	0.932	1	<0.001
GSE55643 (4)	Lunardi S	2014	LAMA3	7	5.49	1.42	45	7.45	1.67	0.005	0.810	0.655	0.964	0.009
			LAMA4	7	12.67	1.14	45	13.07	0.89	0.267	0.508	0.276	0.740	0.947
			LAMB3	8	13.72	1.46	45	15.21	1.52	0.014	0.781	0.636	0.926	0.018
			LAMC2	8	7.72	1.43	45	9.91	1.91	0.025	0.848	0.744	0.951	0.003
GSE56560 (5, 6)	Haider S	2014	LAMA3	7	84.17	21.21	28	263.05	147.33	<0.001	1	1	1	<0.001
			LAMA4	7	173.70	38.43	28	631.87	167.24	<0.001	1	1	1	<0.001
			LAMB3	7	155.92	64.35	28	799.61	503.45	<0.001	0.99	0.964	1	<0.001
			LAMC2	7	100.20	56.12	28	1177.88	622.80	<0.001	1	1	1	<0.001
GSE62165 (7)	Janky R	2016	LAMA3	13	6.05	1.38	118	9.33	1.10	<0.001	0.952	0.889	1	<0.001
			LAMA4	13	6.71	0.71	118	8.73	0.53	<0.001	0.983	0.950	1	<0.001
			LAMB3	13	5.59	1.55	118	9.73	0.91	<0.001	0.980	0.951	1	<0.001
			LAMC2	13	6.48	1.25	118	10.85	0.88	<0.001	0.996	0.989	1	<0.001
GSE62452 (8)	Yang S	2016	LAMA3	61	4.64	0.54	69	5.89	0.82	<0.001	0.899	0.845	0.953	<0.001
			LAMA4	61	6.03	1.18	69	6.83	0.73	<0.001	0.708	0.615	0.800	<0.001
			LAMB3	61	4.38	0.84	69	6.46	1.29	<0.001	0.906	0.857	0.955	<0.001
			LAMC2	61	4.66	1.24	69	7.30	1.43	<0.001	0.909	0.860	0.958	<0.001
GSE91035	Schmittgen T	2016	LAMA3	25	8.79	1.48	25	10.42	1.96	0.002	0.725	0.581	0.869	0.006
			LAMA4	25	8.18	1.12	25	9.63	1.12	<0.001	0.816	0.698	0.934	<0.001
			LAMB3	25	9.78	2.05	25	12.18	2.49	0.001	0.774	0.642	0.907	0.001
			LAMC2	25	9.06	1.82	25	11.77	2.48	<0.001	0.797	0.667	0.926	<0.001
GSE101448 (9)	Klett H	2018	LAMA3	19	8.36	0.83	24	10.41	1.31	<0.001	0.877	0.766	0.988	<0.001
			LAMA4	19	9.53	0.74	24	11.13	0.49	<0.001	0.996	0.984	1	<0.001
			LAMB3	19	9.44	1.21	24	13	1.32	<0.001	0.954	0.889	1	<0.001
			LAMC2	19	8.66	0.74	24	11.56	1.19	<0.001	0.954	0.885	1	<0.001
GSE102238	Sun Y	2017	LAMA3	50	4.16	0.67	50	3.88	0.43	0.015	0.620	0.510	0.730	0.039
			LAMA4	50	11.49	0.83	50	12.16	0.57	<0.001	0.744	0.645	0.844	<0.001
			LAMB3	50	4.60	0.54	50	4.26	0.36	<0.001	0.309	0.204	0.414	0.001
			LAMC2	50	10.57	1.52	50	11.66	0.75	<0.001	0.714	0.605	0.822	<0.001
USA cohort (10)	Hari Kosanam	2013	LAMC2	35	141.71	179.05	50	499.82	326.85	<0.001	0.845	0.759	0.931	<0.001
			CA19-9	35	23.91	42.6	50	2486.0	3804	<0.001	0.863	0.787	0.940	<0.001

						6		6	.24					
German cohort (10)	Hari Kosanam	2013	LAMC2	100	220.23	247.59	100	440.95	344.88	<0.001	0.695	0.621	0.768	<0.001
			CA19-9	100	17.51	64.50	100	602.33	1552.56	<0.001	0.847	0.791	0.903	<0.001
Japan cohort (10)	Hari Kosanam	2013	LAMC2	50	139.91	144.24	50	382.20	311.23	<0.001	0.787	0.695	0.878	<0.001
			CA19-9	50	101.24	611.75	50	1699.73	3096.52	0.001	0.827	0.739	0.914	<0.001

Abbreviations: PDAC, pancreatic ductal adenocarcinoma; SD, standard deviation; AUC, area under the curve; CI, confidence interval.

Supplementary References

1. Zhang G, Schetter A, He P, Funamizu N, Gaedcke J, Ghadimi BM, Ried T, Hassan R, Yfantis HG, Lee DH, Lacy C, Maitra A, Hanna N, et al. DPEP1 inhibits tumor cell invasiveness, enhances chemosensitivity and predicts clinical outcome in pancreatic ductal adenocarcinoma. PLoS One. 2012; 7:e31507. https://doi.org/10.1371/journal.pone.0031507 PMID:22363658

2. Zhang G, He P, Tan H, Budhu A, Gaedcke J, Ghadimi BM, Ried T, Yfantis HG, Lee DH, Maitra A, Hanna N, Alexander HR, Hussain SP. Integration of metabolomics and transcriptomics revealed a fatty acid network exerting growth inhibitory effects in human pancreatic cancer. Clin Cancer Res. 2013; 19:4983–93. https://doi.org/10.1158/1078-0432.CCR-13-0209 PMID:23918603

3. Donahue TR, Tran LM, Hill R, Li Y, Kovochich A, Calvopina JH, Patel SG, Wu N, Hindoyan A, Farrell JJ, Li X, Dawson DW, Wu H. Integrative survival-based molecular profiling of human pancreatic cancer. Clin Cancer Res. 2012; 18:1352–63. https://doi.org/10.1158/1078-0432.CCR-11-1539 PMID:22261810

4. Lunardi S, Jamieson NB, Lim SY, Griffiths KL, Carvalho-Gaspar M, Al-Assar O, Yameen S, Carter RC, McKay CJ, Spoletini G, D'Ugo S, Silva MA, Sansom OJ, et al. IP-10/CXCL10 induction in human pancreatic cancer stroma influences lymphocytes recruitment and correlates with poor survival. Oncotarget. 2014; 5:11064–80. https://doi.org/10.18632/oncotarget.2519 PMID:25415223

5. Haider S, Wang J, Nagano A, Desai A, Arumugam P, Dumartin L, Fitzgibbon J, Hagemann T, Marshall JF, Kocher HM, Crnogorac-Jurcevic T, Scarpa A, Lemoine NR, Chelala C. A multi-gene signature predicts outcome in patients with pancreatic ductal adenocarcinoma. Genome Med. 2014; 6:105. https://doi.org/10.1186/s13073-014-0105-3 PMID:25587357

6. Wang J, Dumartin L, Mafficini A, Ulug P, Sangaralingam A, Alamiry NA, Radon TP, Salvia R, Lawlor RT, Lemoine NR, Scarpa A, Chelala C, Crnogorac-Jurcevic T. Splice variants as novel targets in pancreatic ductal adenocarcinoma. Sci Rep. 2017; 7:2980. https://doi.org/10.1038/s41598-017-03354-z PMID:28592875

7. Janky R, Binda MM, Allemeersch J, Van den Broeck A, Govaere O, Swinnen JV, Roskams T, Aerts S, Topal B. Prognostic relevance of molecular subtypes and master regulators in pancreatic ductal adenocarcinoma. BMC Cancer. 2016; 16:632. https://doi.org/10.1186/s12885-016-2540-6 PMID:27520560

8. Yang S, He P, Wang J, Schetter A, Tang W, Funamizu N, Yanaga K, Uwagawa T, Satoskar AR, Gaedcke J, Bernhardt M, Ghadimi BM, Gaida MM, et al. A novel MIF signaling pathway drives the malignant character of pancreatic cancer by targeting NR3C2. Cancer Res. 2016; 76:3838–50. https://doi.org/10.1158/0008-5472.CAN-15-2841 PMID:27197190

9. Klett H, Fuellgraf H, Levit-Zerdoun E, Hussung S, Kowar S, Küsters S, Bronsert P, Werner M, Wittel U, Fritsch R, Busch H, Boerries M. Identification and Validation of a Diagnostic and Prognostic Multi-Gene Biomarker Panel for Pancreatic Ductal Adenocarcinoma. Front Genet. 2018; 9:108. https://doi.org/10.3389/fgene.2018.00108 PMID:29675033

10. Kosanam H, Prassas I, Chrystoja CC, Soleas I, Chan A, Dimitromanolakis A, Blasutig IM, Rückert F, Gruetzmann R, Pilarsky C, Maekawa M, Brand R, Diamandis EP. Laminin, gamma 2 (LAMC2): a promising new putative pancreatic cancer biomarker identified by proteomic analysis of pancreatic adenocarcinoma tissues. Mol Cell Proteomics. 2013; 12:2820–32.

https://doi.org/10.1074/mcp.M112.023507
PMID:23798558
